# The impact of iron supplementation efficiency in female blood donors with a decreased ferritin level and no anaemia. Rationale and design of a randomised controlled trial: a study protocol

**DOI:** 10.1186/1745-6215-10-4

**Published:** 2009-01-16

**Authors:** Baptiste Pedrazzini, Sophie Waldvogel, Jacques Cornuz, Paul Vaucher, Raphael Bize, Jean-Daniel Tissot, Alain Pecoud, Bernard Favrat

**Affiliations:** 1 Department of Ambulatory Care and Community Medicine, University Hospital of Lausanne (CHUV), University of Lausanne, Lausanne, Switzerland; 2 Blood Transfusion Service of the Swiss Red Cross, Lausanne, Switzerland

## Abstract

**Background:**

There is no recommendation to screen ferritin level in blood donors, even though several studies have noted the high prevalence of iron deficiency after blood donation, particularly among menstruating females. Furthermore, some clinical trials have shown that non-anaemic women with unexplained fatigue may benefit from iron supplementation. Our objective is to determine the clinical effect of iron supplementation on fatigue in female blood donors without anaemia, but with a mean serum ferritin ≤ 30 ng/ml.

**Methods/Design:**

In a double blind randomised controlled trial, we will measure blood count and ferritin level of women under age 50 yr, who donate blood to the University Hospital of Lausanne Blood Transfusion Department, at the time of the donation and after 1 week. One hundred and forty donors with a ferritin level ≤ 30 ng/ml and haemoglobin level ≥ 120 g/l (non-anaemic) a week after the donation will be included in the study and randomised. A one-month course of oral ferrous sulphate (80 mg/day of elemental iron) will be introduced vs. placebo. Self-reported fatigue will be measured using a visual analogue scale. Secondary outcomes are: score of fatigue (Fatigue Severity Scale), maximal aerobic power (Chester Step Test), quality of life (SF-12), and mood disorders (Prime-MD). Haemoglobin and ferritin concentration will be monitored before and after the intervention.

**Discussion:**

Iron deficiency is a potential problem for all blood donors, especially menstruating women. To our knowledge, no other intervention study has yet evaluated the impact of iron supplementation on subjective symptoms after a blood donation.

**Trial registration:**

NCT00689793

## Background

Clinical trials have shown that non-anaemic women with unexplained fatigue may benefit from iron supplementation [1-5]. A one-month ferrous sulphate treatment showed positive effects on fatigue in women with a mean serum ferritin of 30 ng/ml [5]. Improved aerobic capacity, assessed by VO_2max_, has also been associated with iron supplementation in women with non-anaemia iron deficiency [6].

The haemoglobin content of a whole-blood donation (450 ml) is around 55 g to 70 g, and the iron content is around 187 mg to 283 mg. This iron amount corresponds to a range of 66% to 97% of the total stored iron in an average menstruating woman. According European Council directives, it is not mandatory to measure the donor's ferritin level before a blood donation. However, to avoid iron deficiency anaemia induced by blood donations, women's haemoglobin level is controlled, and their blood donation frequencies are limited to three or four per year.

According to a recent survey, 11% of female and 4% of male blood donors complained from fatigue after a donation [7]. The greater proportion of haemoglobin, which is removed from women who are smaller in size and have higher prevalence of iron deficiency, can probably explain the gender difference. Moreover, fatigue is the third complication following a blood donation, after bruise and sore arm. This has an adverse impact on blood donations. Fatigue was associated with a 20% reduction in blood donor return rates at one year post donation [8].

Two recent trials have also shown that a short period of ferrous sulphate supplementation decreased the incidence of iron deficiency in blood donors [9,10]. Furthermore, it produced a rapid improvement in haemoglobin level between blood donations, which improved the fidelity of donors [10]. To our knowledge, no study has shown the impact of iron supplementation on female blood donors' symptoms, such as fatigue.

## Method/Design

### Objectives

Our primary objective is to test the hypothesis that a one-month iron supplementation (oral ferrous sulphate, 80 mg/day of elemental iron), after blood donation, has positive effects on symptoms of fatigue in women blood donors with a serum ferritin concentration ≤ 30 ng/ml, and no anaemia.

Secondary objectives are to assess the effect of treatment on aerobic capacity, mood, quality of life, and haematological parameters.

### Primary outcomes

We will assess changes in self-reported fatigue at one-month follow-up. The self-reported fatigue will be measured using a 10 cm visual analogue scale (VAS), which has demonstrated good psychometric properties in this context [11].

### Secondary outcomes

We will assess changes in fatigue, mood disorders, and quality of life [11-13], using a self-administered questionnaire; changes in maximal aerobic power (estimated by the Chester Step Test) [14]; changes in serum ferritin and haemoglobin; and adherence to treatment.

## Design

**Figure 1:**
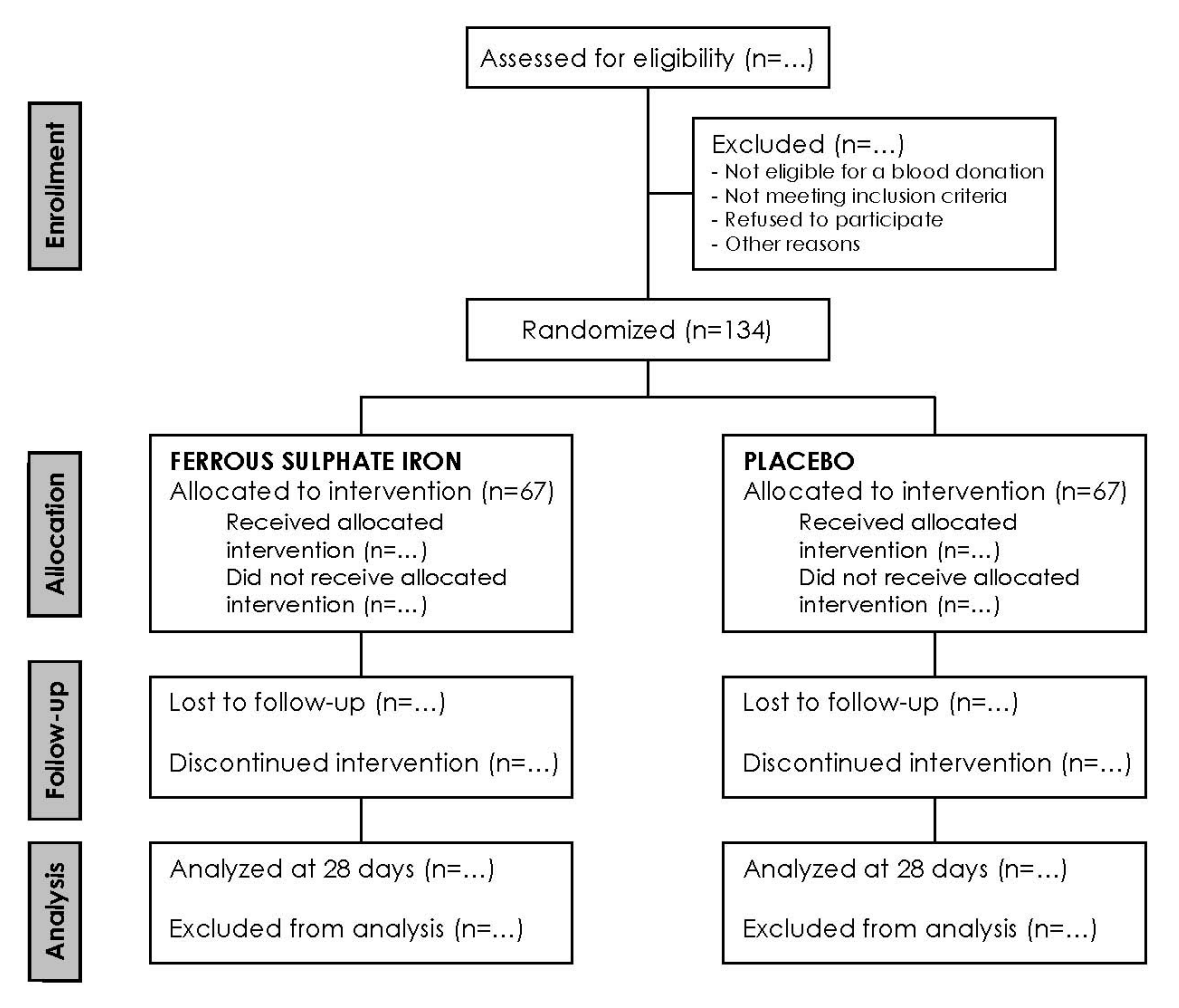
**flow chart**.

Our study will be a pragmatic randomised placebo controlled trial. Donors will join the study and sign an informed consent before blood donation. Approximately 450 ml of venous blood will be collected (not used for the study) within a blood pack set (NGR6449B, Fenwal, Belgium), which allow a predonation sampling of an excess 12 ml for further testing in our study. One week after, volunteers with a concentration of ferritin ≤ 30 ng/ml, and a concentration of haemoglobin ≥ 120 g/l, will be included in the study and randomised to receive either ferrous sulphate (80 mg/day of elemental iron) or placebo for four weeks (See Figure 1).

## Setting

This randomised controlled trial will take place at the University Hospital of Lausanne. The blood donation will take place in the Blood Transfusion Service, and the follow-up in the Department of Ambulatory Care and Community Medicine.

## Study participants

Study participants will be recruited among women coming spontaneously to the Blood Transfusion Service to donate blood. Donors aged 18 yr – 50 yr and eligible to donate blood, according to the blood donation eligibility guidelines of the Swiss Transfusion Service, will be considered for inclusion. Exclusion criteria are psychiatric conditions or diseases that render the participant unable to give consent; thyroid, hepatic, rheumatic, kidney, cardiopulmonary, or intestinal disease; acute or chronic inflammation, diabetes, hemochromatosis, pregnancy, and medical treatment that could perturb iron absorption.

## Randomisation, allocation and masking of the study

A physician working at the Blood Transfusion Service will be responsible for seeing all potential participants (i.e., female donors aged 18 yr – 50 yr) and will assign the next available study number. Once eligibility criteria for a blood donation is fulfilled and an informed consent is obtained, blood donation will be performed. The randomisation will take place a week after the blood donation with the following criteria for inclusion. Haemoglobin level ≥ 120 g/l, ferritin level ≤ 30 ng/ml. Randomisation will take place at an independent pharmacy, according to a pre-established computer-generated list using simple randomisation. Each drug package will be coded with a unique number according to the randomisation schedule. The codes will be held by the pharmacist and remained unbroken until the analyses are completed. A research assistant responsible for seeing the blood donors will allocate the next available treatment pack in a telephone call to the pharmacist. Patients, caregivers, and assessors will be blinded to treatment assignment until the end of the trial. Participants will remain on the same allocation throughout the study (intention-to-treat protocol). An electronic device that assesses adherence to treatment will also be delivered. The pills and electronic device will have the identical appearance for the experimental and placebo groups. The iron supplement and the placebo will have the same taste.

## Treatment

Volunteers will receive either 80 mg/day oral ferrous sulphate (Tardyferon, Robapharm, Boulogne, France) or placebo for four weeks. To decrease side effects, the pills will be taken during breakfast. Currently, oral ferrous sulphate is the reference treatment for iron deficiency without anaemia. Oral ferrous sulphate is resorbed mainly in the duodenum and proximal jejunum. Iron resorption depends directly on the level of hepcidin. Described side effects are upset stomach, constipation, diarrhoea, nausea, and black or dark-coloured stools. No serious complications have been reported. However, uncommon cases of allergic reactions have been described. Volunteers will be asked not to take over the counter vitamin or iron supplements.

## Measurements

Paramedical staff will collect data. Ten cm visual analogue scales, ranging from "no fatigue at all" to "very severe fatigue," associated with another self administered questionnaire focusing on fatigue (Fatigue Severity Scale, [11]) will be completed at randomisation (D0), and after the experimental phase (D28). The initial questionnaire has been translated into French and back translated for verification. The appropriateness and acceptability of the questions have been tested in our setting in pilot studies. For additional outcomes, depression and anxiety symptoms will be assessed using the Prime-MD questionnaire [13]. Health-related quality of life will be measured using the SF-12 questionnaire [12]. An evaluation of the amount of menstrual blood loss (Pictorial Bleeding Assessment Chart, [15]) will also be performed at baseline to detect any possible hypermenorrhea.

Complete blood count and ferritin concentration will be measured at D-7 (day minus 7), D0 and D28. C-reactive protein (CRP), an acute phase protein, will be analysed at D0 to identify volunteers whose ferritin concentration might be falsely elevated by occult infection or inflammation. Complete blood count will be measured using the Sysmex XE 2100 (Roche) haematology analyser. Ferritin and CRP will be measured by immunoturbidimetry.

To assess the change in aerobic capacity at baseline (D0) and after iron treatment (D28), a step test (Chester Step Test) will be carried out. Measurements obtained with this protocol have been demonstrated to give excellent test-retest reliability, and are highly correlated with VO_2max _(r = 0.92) [14].

Adherence to treatment will be measured by an electronic device, Medication Event Monitoring System (MEMS; Aardex Europe, Switzerland), which records the date and time that the pill container is opened [16]. Questions will be asked at D28 to evaluate whether the electronic monitoring was properly used (for example, if each opening is correlated with the consumption of one pill). Unused pills will also be counted. Adherence will be quantified by dividing the number of times the device was opened by the total number of days of observation. A study that used this system has shown that compliance and motivation to take the treatment were improved [17].

The success of blinding will also be evaluated in the questionnaire at D28. Serious adverse events will be reported to the principal investigator within 48 hr, and will be recorded in follow-up notes.

## Statistical methods

### Sample size

The main outcome variable is the level of fatigue at one month. The sample size for randomised volunteers was calculated using a two-sample comparison of means to detect a one point difference between the groups on the visual analogue scale, similar to the minimal clinically appreciable difference for pain [18]. According to a previous study [5], we can expect a standard deviation of two points. For a two-tailed test (α = 0.05, power = 0.80), each group should include 63 participants. Anticipating a 10% dropout rate, the total sample size is rounded to 140 participants.

### Statistical analysis

Analysis will be conducted on an intention-to-treat basis. The measure of effect is the change in fatigue level. The null hypothesis is that there will be no difference in fatigue VAS scores between the experimental and control groups at 28 days, adjusted for the baseline level of fatigue on the same scale. The significance level will be set at 5%, using a linear regression with the treatment group, and using the baseline values of fatigue as independent variables, and using fatigue levels at 28 days as the dependent variables. The measure of effect for the secondary outcomes will be assessed by the same method. If ferritin levels are not normally distributed, a transformation will be used. All calculations will be performed with StataCorp 2008, Statistical Software: Release 10.0, Stata Corporation, College Station, Texas.

### Missing data and drop-outs

Missing data will be handled using multiple imputation. Reasons for dropping out will be compared between the placebo and the ferrous sulphate groups at a significant level of 0.05. In the intention-to-treat analysis, dropouts will be considered as missing data and analysis will use multiple imputation to interpret the results.

## Ethical aspects

The study was approved in July 2008 by the University of Lausanne Ethics Committee for clinical research. The main risk for participants is developing anaemia after the blood donation. The blood test a week after blood donation will permit diagnosis of this complication, and anaemic volunteers will be removed from the study before randomisation. In this case, a doctor from the Blood Transfusion Service will introduce a three-month ferrous sulphate treatment and inform the individual's general practitioner. Another type of risk that may be encountered by volunteers is due to side effects of the treatment. As documented above, side effects of ferrous sulphate are well documented and tolerated by a majority of treated patients. Finally, there is no risk of inducing an iron overload in volunteers because only donors with a ferritin level ≤ 30 ng/ml will receive the treatment.

## Forecast execution dates

We plan to enrol the first donor in November 2008, and the last in March 2009. The study of the last donor should be complete in July 2009, and publication is planned for July 2010.

## Discussion

Iron is an essential nutrient for human cells, which blood donors lose with each donation. Menstruating women are particularly at risk for iron depletion, even before blood donation. Actually, recommendations in force for blood donation do not include screening of ferritin levels of regular donors to ensure that their iron balances are not negative. Also, there is no difference in approach to women of childbearing age and to postmenopausal women, in terms of donation frequency or short-term iron substitution.

Studies have shown that iron-deficiency anaemia is associated with fatigue, which can be partially reversed with iron treatment [1-5]. Moreover, iron supplementation to blood donors has been studied [9,10]. However, outcomes of those trials stressed only the return rates of donors, and improving ferritin or haemoglobin levels at the time of the next donation.

The strength of the present study is that, to our knowledge, this is the first randomised controlled trial that tests iron supplementation efficacy on subjective symptoms, and that measures results during the time between two donations. If our hypothesis is confirmed, ie that post-donation iron supplementation can relieve adverse subjective symptoms related to iron deficiency without anaemia, management of blood donors should be revisited.

## Competing interests

Competing interests: BF has taken part in advisory board meetings and received honoraria to speak at meetings of drug companies producing drugs to treat iron deficiency. The other authors have no competing interest.

## Authors' contributions

BP and SW are the principal researchers, and developed the original idea of the study. BF, JC, AP, PV, RB, and JDT participated in the conception and design of the study. BF and PV developed the statistical method. All authors have read and corrected draft versions and approved the final version.
